# A new fireworm (Amphinomidae) from the Cretaceous of Lebanon identified from three-dimensionally preserved myoanatomy

**DOI:** 10.1186/s12862-015-0541-8

**Published:** 2015-11-17

**Authors:** Luke A. Parry, Paul Wilson, Dan Sykes, Gregory D. Edgecombe, Jakob Vinther

**Affiliations:** Bristol Life Sciences Building, University of Bristol, 24 Tyndall Avenue, Bristol, BS8 1TH UK; Department of Earth Sciences, The Natural History Museum, Cromwell Road, London, SW7 5BD UK; Imaging and Analysis Centre, The Natural History Museum, Cromwell Road, London, SW7 5BD UK

**Keywords:** Annelida, Polychaetes, Amphinomidae, Cretaceous, *Rollinschaeta*, Musculature

## Abstract

**Background:**

*Rollinschaeta myoplena* gen. et sp. nov is described from the Late Cretaceous (Cenomanian) Konservat-Lagerstätten of Hakel and Hjoula, Lebanon. The myoanatomy of the fossils is preserved in exceptional detail in three dimensions as calcium phosphate, allowing the musculature of the body wall, gut and parapodia to be reconstructed in detail.

**Results:**

The major muscle groups of polychaetes can be identified in *Rollinschaeta*, including longitudinal muscle bands, circular muscles, oblique muscles, the parapodial muscle complex and the gut musculature, with a resolution sufficient to preserve individual fibres. To allow meaningful comparison with the phosphatized fossil specimens, extant polychaetes were stained with iodine and visualised using microCT. *Rollinschaeta myoplena* possesses two pairs of dorsal longitudinal muscles, dorsal and ventral circular muscles and a single pair of ventral longitudinal muscles. While six longitudinal muscle bands are known from other polychaete groups, their presence in combination with circular muscles is unique to Amphinomidae, allowing these fossils to be diagnosed to family level based solely on their myoanatomy. The elongate, rectilinear body and equally sized, laterally projecting parapodia of *Rollinschaeta* are found only within Amphinominae, demonstrating that the Cretaceous species is derived amongst Amphinomida.

**Conclusion:**

The uniquely preserved myoanatomy of *Rollinschaeta* has allowed diagnosis of a fossil annelid to subfamily level using microCT as a comparative tool for exploring myoanatomy in fossil and extant polychaetes. Our results demonstrate that fossilized muscles can provide systematically informative anatomical detail and that they should be studied when preserved.

## Background

Although relatively rare, polychaete body fossils provide key insights into the evolution of annelids, such as the sequence of character acquisition in the annelid stem group [1–3], the affinities of enigmatic extinct groups [4] and diverse ancient faunas [5, 6]. Important examples of the latter include the early Cambrian Sirius Passet [1, 7], middle Cambrian Burgess Shale [8], Ordovician Fezuoata Biota [4] and Carboniferous Mazon Creek [5, 6]. Fossil annelids can be found in a wide array of preservational modes and depositional settings, ranging from carbonaceous compressions in offshore marine settings, within ironstone concretions in marginal marine settings [5] and by authigenic mineralisation via pyritisation [4, 9] or phosphatisation [10].

**Table 1:** Details of extant taxa used for CT scanning

Taxon	Family	Locality	Exposure (ms)	X-ray kv	X-ray ua	Voxel size (mm)
*Nothria conchylega*	Onuphidae	Baffin Bay, Canada	500	165	160	0.005
*Eurythoe complanata*	Amphinominae	Puerto de la Cruz, Tenerife, Canary Islands	500	165	160	0.007
*Archinome* cf. *tethyana*	Archinominae	Snake Pit vent field, Mid-Atlantic Ridge	708	100	150	0.009
*Goniada maculata*	Goniadidae	ENE of Deget, off Frederikshavn, N Kattegat, Denmark	354	160	160	0.005
*Glycera alba*	Glyceridae	Frederikshavn, N Kattegat, Denmark	354	160	160	0.005
*Hediste diversicolor*	Nereididae	W of Nibe Bredning, Limfjorden, N. Denmark	354	160	160	0.005
*Harmothoe imbricata*	Polynoidae	Bredefjord, S Greenland, Rink	500	165	160	0.01
*Nephtys hombergii*	Nephtyidae	Kaas Bredning, Limfjorden, N. Denmark	354	160	160	0.005

Despite the preservation of labile tissues in the biotas highlighted above, polychaete fossils are typically classified based on the morphology of recalcitrant structures, such as the hollow, calcareous chaetae of two amphinomid species from the Carboniferous [11], the distinctive chaetal baskets of flabelligerids from Mazon Creek [6], or the presence of distinctive jaws [5, 12]. More rarely, the affinities of fossil polychaetes are determined using the morphology of soft anatomy. Key examples include the preservation of soft parts in machaeridians [4] and the three-dimensionally preserved Palaeozoic polychaetes *Arkonips* [9] and *Kenostrychus* [13].

The polychaete fauna of the Cretaceous Konservat-Lagerstätten of Hakel, Hjoula and Al-Namoura , Lebanon was described by Bracchi and Allessandrello [12], who assigned the fossils to six families with seven genera and 17 species. These taxa are all contained within the orders Phyllodocida and Eunicida and were primarily identified based on jaw morphology. Soft tissues are generally poorly preserved in these fossils, although paired longitudinal muscle bands were highlighted in a single specimen of *Ferragutia cenomania* (Goniadidae) [12]. These two orders represent the bulk of diversity of errant polychaetes, with only two families (Euphrosinidae and Amphinomidae) contained within the third order, Amphinomida. The close relationship between these three orders is well established based on morphological data [14, 15], but is currently uncertain based on phylogenomic data [16].

Herein, we describe a new species of Cretaceous polychaete from Lebanon with extensive preservation of muscle tissue, including the muscles of the body wall, gut and parapodia. Polychaete body fossils that preserve evidence of muscle anatomy are rare and at present are known only from Sirius Passet [7], the Silurian Eramosa biota [17], the Jurassic of Solnhofen [10], the Cretaceous Lägerstatten of Lebanon [12] and a possible annelid from the Wattendorf Plattenkalk [18].

The new species preserves myoanatomy in exquisite detail, including the body wall circular and longitudinal muscles, gut musculature and parapodial muscle complex. This is compared with the myoanatomy of errant polychaetes from the published literature as well as novel data from CT scanning of extant polychaetes. The described myoanatomy is unique to the Amphinomidae and the new taxon, formally named as *Rollinschaeta myoplena* gen. nov. sp. nov., preserves further characters unique to Aciculata and Amphinomida. Due to the exceptional state of preservation of muscles in this taxon, it currently has the best-known myoanatomy of any fossil annelid and perhaps any fossil animal besides those preserved in amber [19].

## Results and discussion

### Preservation

**Fig. 1:**
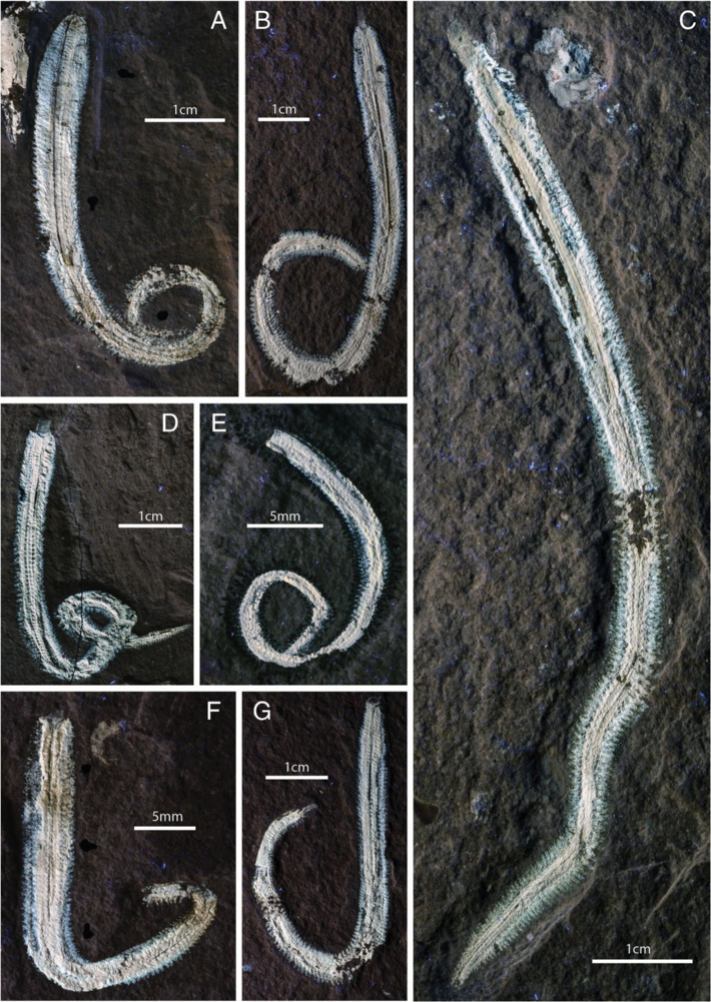
Specimens of *Rollinschaeta myoplena*. **a**) NHMUK PI AN 15074 (holotype); **b**) AN 15072; **c**) AN 15077; **d**) AN 15078; **e**) AN 15066; **f**) AN 15075; **g**) AN 15070. All photographs taken under UV light

**Fig. 2:**
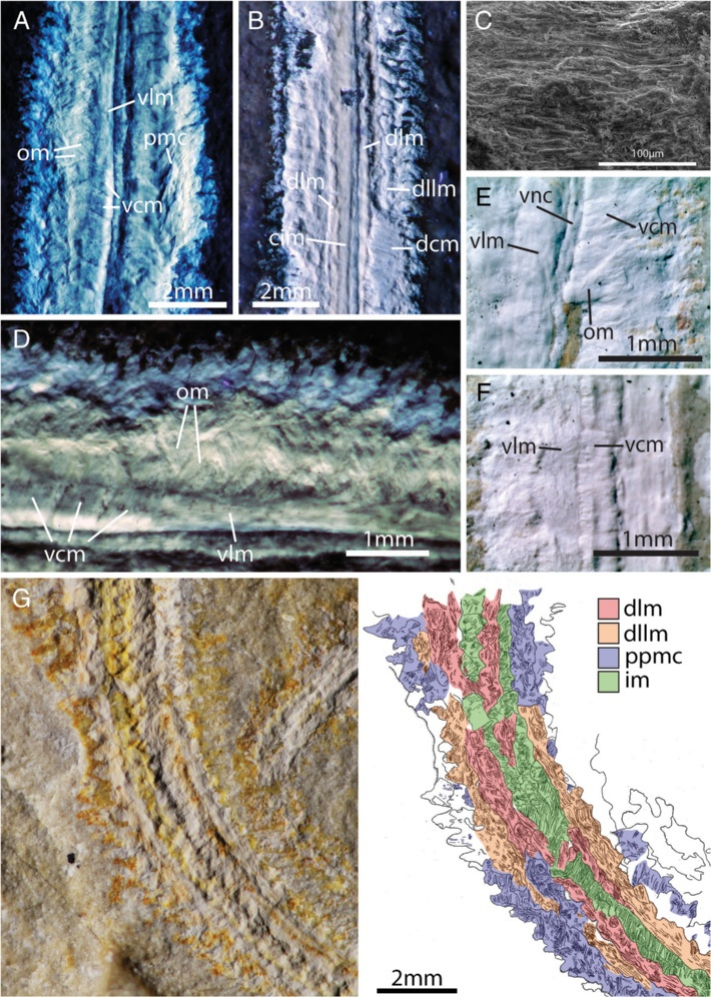
Myoanatomical features of *Rollinschaeta myoplena.* **a**) NHMUK PI AN15070 UV light; **b**) AN15077 UV light; **c**) SEM backscatter; **d**) AN15070 UV light; **e**) AN15068 light microscopic photomicrograph; **f**) AN15068 light microscopic photomicrograph; **g**) AN15074 (holotype), photo under low angle plain light at left, camera lucida drawing at right. Abbreviations: dlm – dorsal longitudinal muscle, dllm – dorsolateral longitudinal muscle, vlm – ventral longitudinal muscle, vcm – ventral circular muscle, dcm – dorsal circular muscle, om – oblique muscle, vnc – ventral nerve cord, cim – circular intestinal muscle

**Fig. 3:**
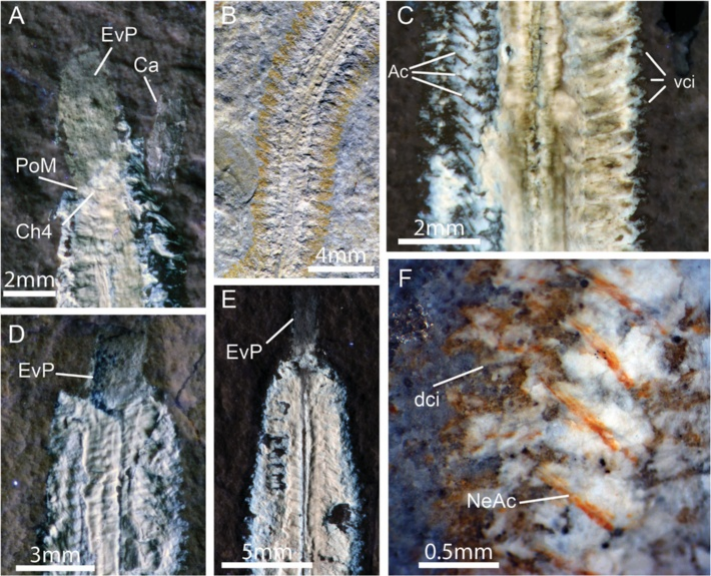
Additional morphological characters of *Rollinschaeta myoplena.* **a**) NHMUK PI AN AN15077 UV light; **b**) AN15077 plain light; **c**) AN15075 UV light; **d**) AN15078 UV light; **e**) AN15072 UV light; **f**) AN15075 light microscopic photomicrograph. Abbreviations: dci – dorsal cirrus, vci – ventral cirrus, PoM – posterior margin of mouth, EvP – everted proboscis, Ch4 – chaetiger 4, Ca – caruncle, Ac – aciculae, NeAc – neuroaciculae
  The arrangement of the anteriormost segments around the head is distinctive, with the posterior of the opening of the buccal cavity occurring at the margin of the third or fourth chaetiger (Fig. 3a). Antennae, palps and cephalised cirri are not preserved. The absence of these structures could be taphonomic given the lack of preservation of non-muscle tissue and cuticle. Musculature had previously been considered absent in the palps of errant annelids [21] and so this could account for their absence, although palp musculature has been identified in Dorvilleidae [22], Nerillidae [23] and Syllidae [24]. Palp muscles are present in the early branching Magelonidae [25] and contractile palps are known from *Canadia spinosa,* a stem group annelid from the Burgess Shale [3, 26], suggesting that palp muscles have undergone multiple independent losses within errant annelids.
- Gut and pharynx
  *Rollinschaeta myoplena* possesses an unarmed, ventral muscular pharynx that opens into a large buccal cavity bounded posteriorly at the fifth chaetiger. The pharynx is eversible, and can be seen at least partly everted in three specimens (Fig. 3a, d and e) in which the buccal cavity is also contracted. The pharynx continues posteriorly into a straight, unbranched gut, preserved as an iron oxide stain with some preservation of gut musculature. The circular musculature of the gut is visible (Fig. 2b), but is poorly preserved compared to the body wall muscles.
- Parapodia
  The parapodia form short outgrowths of the body wall, commonly surrounded by rusty impressions that are sometimes identifiable as individual chaetae (Fig. 3b). The parapodial rami are equal in size and directed laterally from the body. Internally, the parapodia are supported by aciculae, preserved as iron oxide impregnated moulds. The neuropodia are supported by a bundle of ca. 3 aciculae while the notopodial aciculae are singular (Fig. 3f). While cuticular structures are typically poorly preserved, the parapodia rarely preserve short cirriform projections that are likely dorsal and ventral cirri (Fig. 3c and f).

The fossils are preserved in three dimensions as white calcium phosphate in fine-grained sublithographic limestones (Figs. 1 and 2). They are dorso-ventrally compressed and split randomly such that different muscle groups are exposed in different specimens. Preservation is largely limited to muscle tissue. Chaetae are poorly preserved, with aciculae preserved as rust-coloured impressions embedded in the parapodial musculature (Fig. 3c and f), and external chaetae preserved as iron oxide stains along the margins of the parapodia. Preservation of cuticle and external morphological features is absent except for rare projections from the parapodia interpreted as dorsal and ventral parapodial cirri (Fig. 3c and f). Preservation of muscle anatomy in this taxon is pervasive and apparently independent of size, with juvenile specimens only 39 mm long also preserving fine details of muscle anatomy (Fig. 1e). Muscle tissue is sufficiently well preserved that muscle fibres can be identified with the naked eye and light microscopy (e.g. Fig. 2e and f) and SEM (Fig. 2c).

In experiments on polychaete decay, muscles were found to be among the first tissues lost to decay, while external chaetae and aciculae are decay resistant [20]. The exceptional preservation of muscles and poor preservation of chaetae in these polychaetes is therefore in conflict with what is known about character loss during decay in annelids, demonstrating a need to disentangle patterns of decay and preservation in taphonomic studies. The unique and exquisite preservation of muscle tissue is incomparable to any other polychaete taxon known from Hakel/Hjoula, and shows a taxonomic bias acting at the family level. Such a taphonomic bias in myoanatomical preservation has only been previously documented from supraphyletic taxa [10].

### Muscle anatomy

**Fig. 4:**
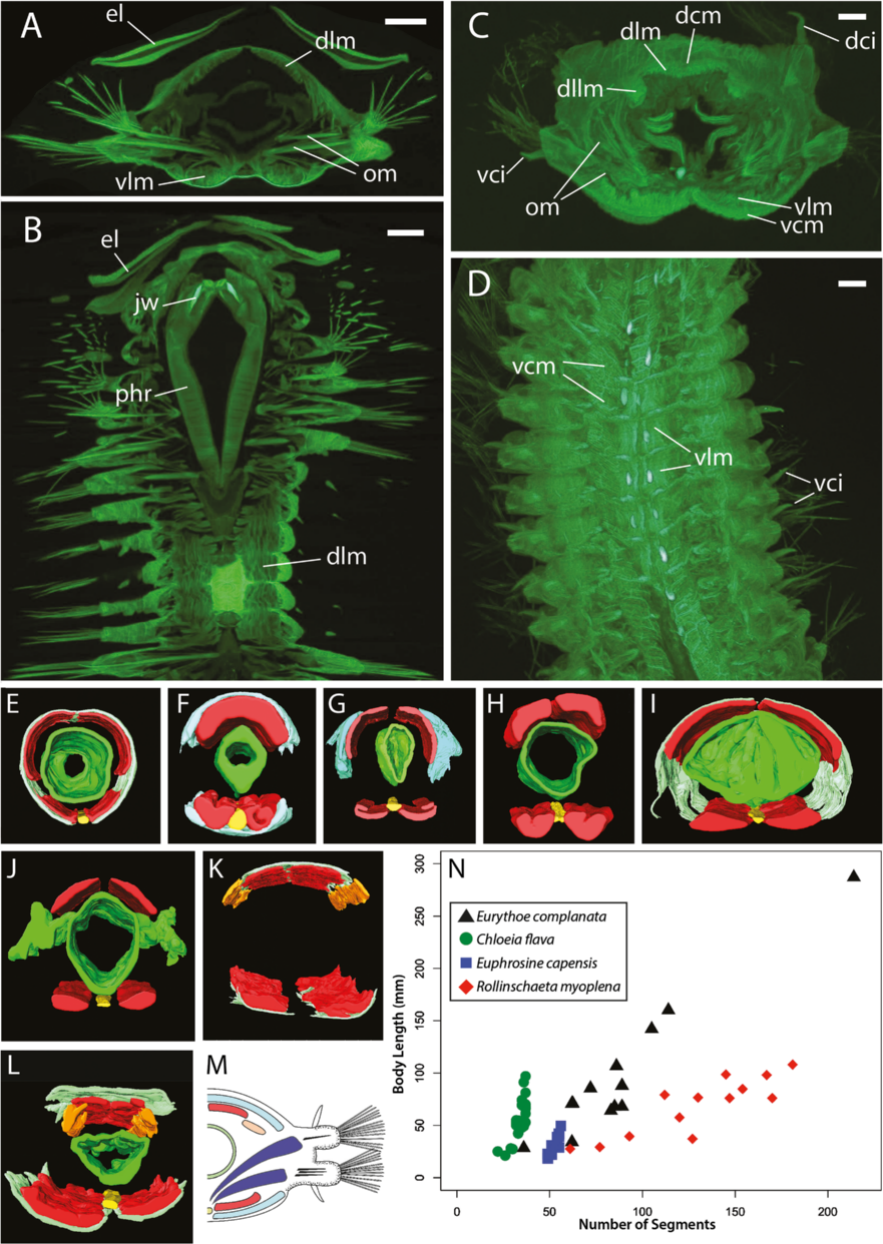
**a**-**b** Virtual histological sections of *Harmothoe imbricata*, scale bars 1 mm. **a** – transverse section, **b** – sagittal section. **c**–**d** virtual histological sections of *Eurythoe complanata,* scale bars 1 mm. **c** – transverse section, **d** – sagittal section. **e**–**l** 3-d models of manually segmented body wall muscles, gut and ventral nerve cord (VNC). Dorsal longitudinal muscles shown in red, dorsolateral longitudinal muscles in orange, circular muscles in blue, VNC in yellow and intestine in green. **e** – *Glycera alba*, **f** – *Goniada maculata*, **g** – *Hediste diversicolor*, **h** – *Nephtys hombergii*, **i** – *Nothria conchylega*, **j** – *Harmothoe imbricata*, **k** – Amphinomidae sp. (intestine and VNC not shown), **l** – *Eurythoe complanata*. **m** – schematic reconstruction of *Rollinschaeta myoplena*, colour scheme as per E-L, with oblique muscles shown in dark blue. **n** – plot of segment number and body length for three extant amphinomid taxa and *Rollinschaeta.* Abbreviations: el – elytrae, dlm – dorsal longitudinal muscle, dllm – dorsolateral longitudinal muscle, vlm – ventral longitudinal muscle, vcm – ventral circular muscle, dcm – dorsal circular muscle, om – oblique muscle, phr – pharynx, jw – jaw, dci – dorsal cirrus, vci – ventral cirrus

- Body wall muscles
  The body wall musculature is composed of dorsal and ventral circular muscle bands, with paired dorsal and ventral longitudinal muscle bands. The ventral circular muscle is broken at the midline at the ventral nerve cord, extends to the base of the neuropodia, and is separated into distinct segmental muscle blocks that terminate at segment boundaries (Fig. 2a, d and f). These muscles are overlain by paired longitudinal muscles that are less laterally extensive than the ventral circular muscles, covering approximately half of their width.
  The dorsal body wall musculature is composed of paired dorsal longitudinal muscles and adjacent and narrower dorso-lateral longitudinal muscles (Fig. 2b and g). These are overlain by a thin sheet of circular muscle that is poorly and discontinuously preserved (Fig. 2g). There is a break in slope between the dorso-lateral and dorsal longitudinal muscles (Fig. 2g), suggesting that the former at least partially underlie the latter. A schematic reconstruction of the myoanatomy is shown in Fig. 4m.
- Oblique and parapodial muscles
  The parapodial muscle complex is an elaborate system of muscles that enables the parapodia to perform a range of movements and consequently is difficult to characterise in *Rollinschaeta* due to compaction and the superposition of muscles associated with each ramus. However, it is possible to observe overlapping portions of musculature associated with their respective rami (Fig. 2d) and rare occurrences of parapodial muscles originating at the midline in association with the ventral nerve cord (Fig. 2e)

### Other anatomical characters

- Gross anatomy
  The body of *Rollinschaeta myoplena* numbers up to ~180 segments in the largest specimens, tapering gently towards the pygidium (Fig. 1). The number of chaetigers covaries with the total length of the body suggesting that growth is indeterminate and that segments are added continuously through life. Taper towards the head is less pronounced, with the maximum width posterior of the head, the exact position depending on the state of contraction of the anterior portion of the animal.
- Head
  Specimen AN 15077 preserves a small area of soft tissue that fluoresces under UV light (Fig. 3a). In this specimen, the head is preserved oblique to bedding, so that the head is preserved in lateral aspect. This piece of tissue is therefore dorsal of the everted pharynx and dorsal body wall. Its position and overall appearance is reminiscent of a caruncle. Preservation is poor, however, and it does not preserve any diagnostic morphological details.

### Comparison with extant polychaetes

The presence of aciculae and well developed parapodial lobes with cirri in *Rollinschaeta* clearly invites comparison with living aciculate polychaetes. While internalised supporting chaetae are known from other annelid taxa, such as Apistobranchidae, Chaetopteridae and Orbiniidae [27], the homology of these chaetae with ‘true’ aciculae is uncertain and they do not co-occur with dorsal and ventral cirri outside of Aciculata [28]. We CT scannned extant polychaetes (Table 1) in order to make meaningful myoanatomical comparisons with *Rollinschaeta* (Fig. 4). Our data suggest that myoanatomy is diagnostic at family level for the groups analysed, with closely related taxa such as Goniadidae and Glyceridae (Glyceriformia) having distinctive arrangement of the body wall muscles (Fig. 4e and f).

While annelids are classically considered to possess an outer layer of circular muscle, this is restricted to Clitellata, certain families within Sedentaria (such as Maldanidae and Capitellidae) and Glyceridae (Fig. 4e), in which the outer transversal fibers are interrupted only at the ventral nerve cord [29]. In other families, circular muscles may be poorly developed, form dorsal and/or ventral semicircular layers, or may be absent entirely [29, 30]. Consequently, the homology of these ‘semicircular’ muscles with true circular muscles is uncertain [29, 31]. Amongst polychaetes, single pairs of dorsal and ventral longitudinal muscles are widespread, occurring in both errant and sedentary taxa [31].

The possession of two pairs of dorsal longitudinal muscles in *Rollinschaeta* is therefore unusual, with such a muscle arrangement only described from Amphinomidae [32] (Fig. 4c, k and l), Polynoidae, Aphroditidae and Chrysopetalidae [31].

Polynoidae and Aphroditidae form part of Aphroditiformia, a group of phyllodocidans with dorsal elytrae, and four dorso-ventral acting jaw elements. Dorsolateral longitudinal muscles are not visible in our CT data for *Harmothoe imbricata* (Polynoidae)*,* circular muscles are absent and the gut contains distinct diverticular pockets (Fig. 4a, b and j). Chrysopetalidae possess paired lateral jaws as well as distinctive paleae on notopodial ridges. Like scaleworms, with which they may be closely related [14], Chrysopetalidae lack circular muscles and possess dorsolateral longitudinal muscles [31, 33]. The absence of jaws, elytrae, gut diverticulae, paleae and tentacular cirri in *Rollinschaeta* suggest that these taxa are not closely related, as is further supported by the presence of circular muscles in *Rollinschaeta.*

*Rollinschaeta myoplena* possesses a suite of morphological characters that are shared with Amphinomida. Specifically, the presence of four dorsal and two ventral longitudinal muscle bands in combination with circular muscles is currently only documented from extant Amphinomidae, while there are at present no data on the myoanatomy of Euphrosinidae, their sister group. The placement of the posterior margin of the buccal cavity at an anterior segment is also shared with amphinomids, with the mouth extending to chaetiger 5 in *Hermodice* [34] and chaetiger 3 in *Chloeia flava* (pers. obs., NHMUK collection). Parapodia with equally sized rami are also an unusual character among Aciculata, known from taxa such as Nephtyidae and only some members of Amphinomidae [14]. The myoanatomy of Nephtyidae is distinct from that of Amphinomidae and *Rollinschaeta* in that nephtyids lack circular muscles and possess only a single pair of dorsal longitudinal muscles (Fig. 4h). Dorsolateral longitudinal muscles are also documented in Sphaerodoridae, but unlike *Rollinschaeta* this taxon lacks typical circular muscles and possesses uniramous parapodia [15, 24, 35].

### Comparison with fossil and extant Amphinomida

Amphinomids have been described as fossils from the Carboniferous in two taxa, *Rhaphidiophorus hystrix* [5] and *Paleocampa anthrax* [11]. Owing to the presence of longitudinally striated chaetae, *Rhaphidiophorus* has been considered a synonym of *Paleocampa* as this character is otherwise unknown among polychaetes [11]. Equally sized parapodial rami are also known from the Silurian aciculate polychaete *Kenostrychus,* but this taxon is currently considered a primitive phyllodocidan [13].

The internal phylogeny of extant Amphinomidae is well characterised, with genera partitioned into two distinct clades [36, 37]. Amphinominae contains taxa with a grossly rectilinear body plan such as *Hermodice* and *Eurythoe* whereas Archinominae contains spindle-shaped genera such as *Chloeia* and *Archinome.* While previous authors have been hesitant to identify the presence of a rectilinear body shape as apomorphic for Amphinominae [36], a fusiform body shape is present in Euphrosinidae, the sister group of Amphinomidae, as well as the oldest fossil amphinomids from the Carboniferous. This suggests that the fusiform body shape is plesiomorphic, although the phylogenetic placement of *Rhaphidiophorus* and *Paleocampa* relative to living taxa is as yet unresolved, since they only preserve amphinomidan plesiomorphies such as calcareous chaetae [11].

The rectilinear morphology of *Rollinschaeta* is therefore consistent with an affinity with Amphinominae, supporting a position nested within the Amphinomidae. This is corroborated by comparisons of segment addition and growth with extant amphinomids and euphrosinids. Segment number is fixed in several fusiform species whereas in rectilinear taxa (including *Rollinschaeta*) segments are added continuously throughout life. For example in *Chloeia flava* segment number is fixed at 37 [38], after which the animal increases in size without the addition of further segments (Fig. 4n) and segment constancy is also known from the fossil *Paleocampa* [11]. An indeterminate segment number is known for *Cryptonome*, a member of the Amphinominae [37], and a linear dependence of segment number on body size is indicated in Fig. 4 for both *Rollinschaeta* and *Eurythoe*, with both taxa adding in excess of 150 segments.

### Systematic palaeontology

This published work and the nomenclatural acts it contains have been registered in ZooBank: http://zoobank.org/urn:lsid:zoobank.org:pub:9122F602-F4CD-4431-A5BA-FE31FB41D24A.

Phylum: Annelida Lamarck, 1809

Subclass: Aciculata Rouse and Fauchald, 1997

Family: Amphinomidae

Subfamily: Amphinominae

Genus: *Rollinschaeta* gen. nov.

*Derivation of name*: Rollins – for Henry Rollins + *chaeta*, from Late Latin, for a bristle, seta or long hair.

*Diagnosis*: Body ranges from 27 to 108 mm in length with 61 to 181 segments tapering gently towards a small pygidium and markedly from anterior chaetigers towards prostomium. Parapodia well developed, biramous with cirri at base of each ramus. Rami approximately equal in size. Aciculae singular in notopodium and present in bundles of ca. 3 in neuropodium. External parapodial chaetae poorly preserved but capillary chaetae present. Pharynx muscular and eversible, with posterior margin of mouth abutting chaetiger 3. Caruncle possibly present. Head appendages including lateral and median antennae and palps, if present, not preserved. Paired dorsal, dorsolateral and ventral longitudinal muscle bands present. Ventral circular muscles present, discontinuous across segment boundaries. Dorsal circular muscles present.

Species: *Rollinschaeta myoplena* gen. nov. sp. nov.

*Derivation of name*: *myo*, from Classical Greek, for muscle + *plenus*, from Latin, for plump or filled.

*Diagnosis*: as for genus.

*Holotype:* NHMUK PI AN 15074, from Hjoula.

*Type locality:* A quarry northwest of the town of Hjoula, Lebanon, 34°07′59.43″N 35°44′39.42″E.

*Paratypes:* 13 specimens, NHMUK PI AN 15075, AN 15076 (type locality, Hjoula), AN 15061, AN 15066, AN 15067, AN 15068, AN 15070, AN 15071, AN 15072, AN 15073, AN 15077, AN 15078, AN 15079, all from Hakel.

## Conclusions

We describe in this paper an amphinomid polychaete from the Late Cretaceous (Cenomanian) deposits of Hakel and Hjoula, Lebanon. To our knowledge, this is the first time an organism could be diagnosed exclusively by preserved myoanatomy. Fossil muscle tissue has largely been considered a taphonomic curiosity and has rarely figured in evolutionary studies [39]. While well-preserved myoanatomy is rare, it is likely to provide novel information about ancient body plans and their functional morphology and should be studied in detail when available. Furthermore we demonstrate that microCT is a fast, non-destructive and effective method for making meaningful myoanatomical comparisons between extant and fossil taxa.

## Methods

A total of 14 specimens from the Late Cretaceous (Cenomanian) limestones of Hakel and Hjoula, northwest Lebanon, were examined (see type material for registrations). All were collected from privately-owned quarries and were acquired by transfer of title. Specimens were illuminated using low angle lighting and under UV to enhance the contrast between the specimen and matrix and highlight muscle groups. In order to allow comparison of the myoanatomy of *Rollinschaeta* with extant polychaetes, specimens were micro-CT scanned. These were first stained by submersion in 1 % iodine in 70 % EtOH. The specimen was dehydrated through a graded alcohol series to 100 % EtOH and then chemically dried using Hexamethyldisilazane (HMDS) to enhance contrast [40]. The dried specimens were then micro-CT scanned with a Nikon HMX ST 225 system, housed in the Natural History Museum. 3,142 projections were collected for each scan and reconstructed using a modified Feldkamp back-projection algorithm [41] in CT Pro (Nikon Metrology, Tring, UK). The data were then manually segmented and visualised using Avizo 8.0 (FEI). Segment counts and approximate measurements of body length were taken from the extant Amphinomida *Euphrosine capensis* (Euphrosinidae) and *Chloeia flava* and *Eurythoe complanata* (Amphinomidae) in order to compare segment addition, growth and gross anatomy with *Rollinschaeta.*

Fossil specimens were imaged a Nikon D90 and Nikon AF-S VR Micro-Nikkor 105 mm f/2 IF-ED lens (with or without a 2x teleconverter) using UV and plain light as well as light microscopy to generate high resolution images and maximise contrast between different muscle groups. Interpretive drawings were made using a camera lucida.
